# Cancer Immunotherapy Based on Natural Killer Cells: Current Progress and New Opportunities

**DOI:** 10.3389/fimmu.2019.01205

**Published:** 2019-05-31

**Authors:** Weilei Hu, Guosheng Wang, Dongsheng Huang, Meihua Sui, Yibing Xu

**Affiliations:** ^1^Institute of Translational Medicine, Zhejiang University School of Medicine, Hangzhou, China; ^2^Department of Surgery & Clinical Research Institute of Zhejiang Provincial People's Hospital, People's Hospital of Hangzhou Medical College, Hangzhou, China; ^3^Center for Cancer Biology and Innovative Therapeutics, Key Laboratory of Tumor Molecular Diagnosis and Individualized Medicine of Zhejiang Province, Hangzhou, China

**Keywords:** cancer immunotherapy, natural killer cell, cytokines, antibodies, adoptive cellular immunotherapy, chimeric antigen receptor, extracellular vesicles

## Abstract

Cancer immunotherapy has been firmly established as a new milestone for cancer therapy, with the development of multiple immune cells as therapeutic tools. Natural killer (NK) cells are innate immune cells endowed with potent cytolytic activity against tumors, and meanwhile act as regulatory cells for the immune system. The efficacy of NK cell-mediated immunotherapy can be enhanced by immune stimulants such as cytokines and antibodies, and adoptive transfer of activated NK cells expanded *ex vivo*. In addition, NK cells can arm themselves with chimeric antigen receptors (CARs), which may greatly enhance their anti-tumor activity. Most recently, extracellular vesicles (EVs) derived from NK cells show promising anti-tumor effects in preclinical studies. Herein, we carefully review the current progress in these NK cell-based immunotherapeutic strategies (NK cells combined with stimulants, adoptive transfer of NK cells, CAR-NK cells, and NK EVs) for the treatment of cancers, and discussed the challenges and opportunities for opening a new horizon for cancer immunotherapy.

## Introduction

Cancer immunotherapy, which works by activating the body's own immune system, has become an increasingly important treatment option for cancers. In recent years, successes in anti-tumor treatments with antibodies and cell-based immunotherapy has become landmark events in the history of tumor treatment (1, 2). As innate immune cells, natural killer (NK) cells are unique and play pivotal functions in cancer immune surveillance. NK cells can eliminate a variety of abnormal or stressed cells without prior sensitization, and even preferentially kill stem-like cells or cancer stem cells (3–5). Upon forming immune synapses with target cells, NK cells release preformed cytolytic granules, including perforin, and granzymes, of which function is to induce cell lysis. Several studies have successfully exploited adoptive transfer of NK cells against various tumors, especially hematological malignancies.

However, cancers employ various tactics to delay, alter, or even stop anti-tumor immunity, leading to failures in the control of tumor growth. The anti-tumor response of NK cells also faces a lot of limitations. First, the poor ability of NK cells to reach tumor tissues limits their application as therapies for solid tumors. This is a common problem of cellular immunotherapy strategies (6). Second, changes in NK cell-activating receptors and their ligands in tumors, may lead to a decreased therapeutic response and tumor progression (7). For example, high levels of NKG2D (Natural-killer Group 2, Member D) ligands are detected in the early stages of colorectal cancer, but their expression decreases as the disease progresses (8). Third, the tumor microenvironment (TME) remains a major barrier to the effectiveness of adoptively transferred NK cells. For example, tumor-infiltrating immune cells such as dendritic cells (DCs), suppressive or tolerogenic macrophages and regulatory T (Treg) cells as well as cancer-associated fibroblasts, which are embedded in the extracellular matrix, may meddle in NK cell activation either through secretion of immunosuppressive cytokines or by interfering with receptor expression (9, 10). For instance, in TEM, TGF-β is recognized as a main inhibitory cytokine of NK cells which limits the number and anti-metastatic function of NK cells. Other factors, such as prostaglandin E2, adenosine or indoleamine 2,3-dioxygenase, can also block the cytotoxic activity of NK cells by, respectively, inducing myeloid-derived supressor cells (11), binding to adenosine A2A receptors expressed on NK cells (12) or catalyzing tryptophan-producing L-kynurenine to inhibit the expression of NKp46 and NKG2D on NK cells (7, 13). In addition, the cytotoxicity of NK cell was found to be inhibited by activated-platelets in the malignant milieu via a series of mechanisms, including the shed and transfer of their MHC class I molecules to tumor cells; undermining NK cell effector function via platelet-derived TGF-β (14) or shielding tumor cells from NK cell attack (15–17).

In order to overcome the above problems many strategies have been explored, either by adding immune stimulants to produce synergistic effects, adoptive transfer of NK cells expanded *in vitro*, or by genetically modifying NK cells themselves to be stronger and more resilient. In addition, nano-vesicle structures secreted by NK cells known as extracellular vesicles (EVs) come into the spotlight for their applications in cancer therapies.

## Biology of NK Cells

NK cells, first identified in 1975, are innate effector lymphocytes that differ from T cells and B cells. NK cells can recognize and kill abnormal cells that lack MHC restriction or prior sensitization, and are considered as the most effective immune cell subpopulation to monitor and clear diseased cells *in vivo* (18, 19).

NK cells are developed from common lymphoid progenitor cells in the bone marrow (BM) and exist within primary and secondary lymphoid tissue, as well as within non-lymphoid tissue including the lungs, liver, and the peripheral blood (PB) (20). Of all the circulating lymphocytes, 10–15% are considered to be NK cells. In humans these cells are CD3^−^CD56^+^ lymphocytes, and in mice they are phenotypically CD3^−^NK1.1^+^. Human NK cells have been identified into two distinct subpopulations based on the density of CD56 on cell surface, as follows: CD56^bright^ CD16^dim^ NK cells, also known as immature NK cells, are cytokine producers especially interferon gamma (IFNγ), which play an important role in immunomodulation; CD56^dim^CD16^bright^ NK cells, known as mature NK cells, are the majority (90%) of NK cells in PB and play significant roles in mediating the immune function of NK cells (21).

NK cells play a key role in the immune innate defense systems to destroy a variety of abnormal or stressed cells (3, 4). Different from other lymphocytes, NK cell recognition is not controlled by antigen specificity but rather through the integration of signals from activating and inhibitory receptors, which are recruited by ligands expressed on putative target cells. The inhibitory receptors which can identify human leukocyte antigen class I (HLA-I) or class I like molecules encompass two distinct classes: the killer immunoglobulin-like receptors (KIR2DL and KIR3DL), and C-type lectin receptors CD94/NKG2A/B (22–24). Because HLA-I are almost expressed on all nucleated cells, the inhibitory response, following recruitment with inhibitory receptors, serves as a recognition of “self,” which dampens NK cell activation and prevent “self” lytic attack (25). Furthermore, programmed death-1 (PD-1), cytotoxic T-lymphocyte-associated protein 4 (CTLA-4), T cell immunoglobulin and mucin domain containing-3 (TIM-3), as well as T cell immunoreceptor with Ig and ITIM domains (TIGIT), act as a series of immune checkpoints and also transmit inhibitory signals when binding to their ligands. The elimination of abnormal cells is enhanced by a lack of constitutive self HLA-I. Stressed and abnormal cell recognition occurs by the stimulation of their cell surface receptors such as: KIRs (KIR2DS and KIR3DS), NKG2D, DNAX Accessory Molecule-1 (DNAM-1), killer cell C-type lectin receptor complex CD94/NKG2C, and natural cytotoxicity receptors (NKp30, NKp44, NKp46) (25).

Direct cytotoxicity for target cells by NK cells is thought to critically rely on cytolytic granules such as perforin and granzymes (26). The death receptor (DR) mediated apoptotic process of abnormal or stressed cells is also a way of direct killing. The caspase enzymatic cascade induced apoptosis is triggered by the interaction between DRs (e.g., FasL, TRAIL) expressed on NK cells and their ligands on target cells (27). Another direct killing mechanism involves antibody dependent cell-mediated cytotoxicity (ADCC) (19). ADCC is usually mediated by immunoglobulin G (IgG) in humans. The Fab moiety and the Fc moiety of the antibody bind to the tumor-associated antigens (TAAs) on tumor cell and CD16A (FcγRIIIA), the activating receptor expressed on NK cell, respectively, to form an immunological synapse between the two. Then, NK cells will be activated and secrete cytotoxic granules to kill tumor cells (For more detailed information about ADCC of NK cells, see section Use of tumor-specific antibodies to mediate ADCC of NK cells). Furthermore, NK cells can function through an indirect way by producing chemokines and cytokines to kill abnormal cells and regulate innate and acquired immune responses (28).

## Immune-stimulatory Molecules to Boost the Anti-tumor Activity of NK Cells

**Figure 1:**
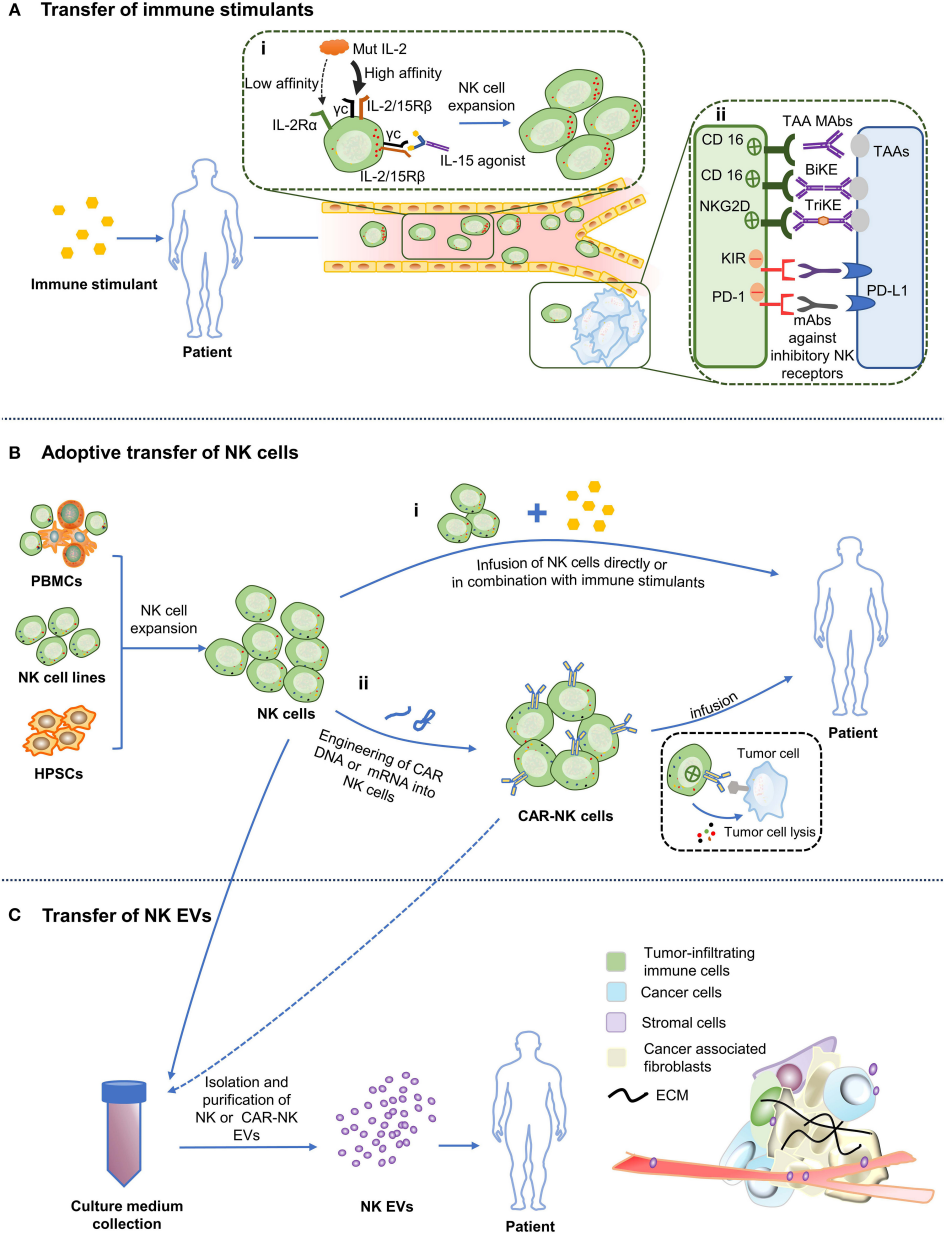
Various NK cell-based immunotherapy approaches. **(A)** Administration of stimulatory cytokines and antibodies to patients triggers activation and expansion of the autologous NK cells and enhance their cytotoxicity. (i) Cytokine. “Super” agonist of IL-2 improves its affinity for IL-2/15Rβ. Arrow width indicates expected intensity of IL-2 signaling. The “super” agonist of IL-15 mimics the physiological trans-presentation of IL-15 to NK cells without the involvement of antigen-presenting cells. (ii) Antibodies. Binding of CD16 to the Fc portion of TAA mAbs leads to NK cells activation and ADCC. Application of BiKE or TriKE target to CD16 or NKG2D (on NK cells) and tumor antigens promotes the formation of immune synapses between NK cells and tumor cells. mAbs against inhibitory receptors on NK cells facilitate NK cytotoxicity. **(B)** Adoptive transfer of NK cells. (i) NK cells obtained from PBMCs, NK cell lines or hPSCs can be infused into patients directly or in conjunction with immune stimulants. (ii) NK cells are designed to express chimeric antigen receptors (CAR), which are then allowed to expand *ex vivo* before being transfused back into the patient. **(C)** Infusion of engineered NK cells and NK/CAR-NK cell derived EVs. Culture medium of expanded NK cells or CAR NK cells can be exploited to isolate EVs and then infused into the patient. TAA, Tumor-associated antigen; PD-L1, Programmed death ligand-1; ECM, Extracellular matrix.

Endogenous NK cells in cancer patients usually have an impaired function because of the alteration of a repertoire of receptors in the cells. This may involve downregulation of activating receptors and/or upregulation of inhibitory receptors (7). Thus, the primary method in immunotherapy treatments is to “push” for immune activation by including additives like cytokines and antibodies that help modulate the mechanisms that improve the quantity and/or quality of the anti-tumor immune response (Figure 1A) (29).

### Cytokines to Argument NK Cell Activity

Cytokines promote the survival, proliferation, differentiation and activation of lymphocytes. Interleukin (IL)−2,−15,−12,−21 as well as−18 improve anti-tumor function of NK cells and boost their proliferation *in vitro* and *in vivo* (30, 31). Here, we focus on the first two. A more detailed review can be found in Fang et al. (32), Lin and Leonard (33).

IL-2 was initially discovered as a T-cell growth factor more than 30 years ago and now is one of the most popular cytokines used to boost cytotoxicity of NK cells (30). However, a high-dose IL-2 therapy can lead to severe adverse effects, including vascular leakage and organ injury caused by activation of the vascular endothelium, where the IL-2 high affinity receptor, IL-2Rα*βγ*, is expressed (34). Furthermore, Treg cells also express high-affinity IL-2Rα*βγ* receptors and are preferentially activated by IL-2, inhibiting NK cell proliferation and cytotoxicity (35). These resulted in the development of alternative forms of IL-2. A new mutant of IL-2 called “super-2” has been constructed with increased affinity for the IL-2/15Rβ subunit present on NK cells, but lower affinity for IL-2Rα subunit (36). Alternatively, variants of IL-2, such as F42K, show decreased affinity *in vitro* for IL-2Rα (37). Another strategy is to create a recombinant fusion protein comprised of a cowpox virus encoded NKG2D binding protein with a mutated form of IL-2 that preferentially and potently stimulates IL-2 on cells bearing NKG2D only, without broadly activating IL-2Rα-bearing cells or inducing side effects in animal models (38).

A superior alternative to IL-2 is IL-15, which preferentially stimulates memory CD8^+^ T cells, and immature and mature NK cells. IL-15 receptors consist of IL-15Rα, IL-2/15Rβ, and γc. IL15Rα expressed on antigen-presenting cells such as DCs and monocytes can present IL-15 *in trans* to IL-2Rβγc receptors expressed on NK and CD8^+^ T cells without activating Tregs (39). IL-15 is the principal γc family cytokine in the cytotoxicity, homeostasis and development of NK cells (30). In the first clinical trial where recombinant human (rh) IL-15 was infused to patients with metastatic malignancies, NK cell proliferation was observed in patients (40). Although two patients showed the clearance of pulmonary lesions, there were no objective responses. In addition, the clinical use of rhIL-15 might be compromised by its short half-life and poorly tolerated by patients (MTD: 0.3 μg/kg/day). Interestingly, rhIL-15 was found to modulate the homeostasis of NK cells, memory CD8^+^ T cells and γδ T cells, but not Treg cells (40). Moreover, a most recent phase I clinical trial reported that subcutaneous administration of rhIL-15 significantly elevated the tolerated dose of rhIL-15 in patients with refractory solid tumor (MTD: 3 μg/kg/day). Subcutaneous administration of rhIL-15 remarkably promoted the proliferation of circulating NK cells and CD8^+^ T cells, especially CD56^bright^ NK cells (41). The biological function of rhIL-15 allows it to be used in combination with other modalities. Indeed, there are several clinical trials (NCT03759184: obinutuzumab; NCT02689453: alemtuzumab; NCT03388632: nivolumab and ipilimumab; NCT03905135: avelumab) underway investigating rhIL-15 in combination with antibodies.

ALT-803 is a superagonist of IL-15 comprising an IL-15Ra fused to IgG1Fc which is bounded to IL-15 mutein (N72D). This special compound is designed to simulate the typical *trans* presentation of IL-15 and has a much longer half-life (25 h vs. < 40 min), which may lead to enhanced ADCC (42, 43). In the first phase I clinical study with ALT-803, 33 patients who relapsed after allogeneic hematopoietic cell transplantation (allo-HCT) received ALT-803 intravenously or subcutaneously. ALT-803 is generally well-tolerated, as no severe dose-limiting toxicities (DLT) and graft-vs.-host disease (GVHD) were observed in both cohorts. Nineteen percent of the subjects showed clinical benefit, with 1 complete remission lasted for 7 months. All patients in this study exhibited increased levels of circulating CD56^bright^NK cells and CD8^+^ T cells, especially those administered subcutaneously (44). Considering its prolonged half-life and potent activating power for CD8^+^ T and NK cells, ALT-803 is being evaluated in a range of ongoing clinical studies in combination with antibodies (NCT02523469: nivolumab, NCT02384954: rituximab) or adoptive transfer of NK cells (NCT01898793, NCT02782546). In a recent Phase 1b clinical trial, ALT-803 combined with anti-PD-1 antibody nivolumab was well-tolerated in non-small cell lung cancer (NSCLC) patients, and 6/21 of patients showed an objective response. Notably, among 11 subjects previously treated with nivolumab alone and relapsed, 3 subjects exhibited partial response, suggesting the addition of ALT-803 may reinforce the anti-tumor activity of antibodies (45). In clinical studies combining ALT-803 with adoptive transfer of NK cells (NCT01898793, NCT02782546), subcutaneously injected ALT-803 is expected to enhance the *in vivo* activation, survival and expansion of donor derived NK cells without severe adverse events (46). Given the fact that ALT-803 has significant anti-tumor activity and did not aggravate side effects associated with nivolumab or Bacillus Calmette Guerin (BCG) (47), meaningful results are promising to be obtained in these underway trials.

### Antibodies to Redirect NK Cytotoxicity

Monoclonal antibodies (mAbs) possess strong inherent efficacy that suits cancer immunotherapy as they have been shown to directly and/or indirectly promote the roles of NK cell *in vivo*. In this section we will discuss tumor-specific antibodies, specific killer engagers and antibodies targeted inhibitory receptors (on NK cells).

#### Use of Tumor-Specific Antibodies to Mediate ADCC of NK Cells

Tumor-specific antibodies are mAbs that work partially by promoting NK cell ADCC through the binding of the IgG Fc part and its activating receptor CD16A (FcγRIIIA) expressed on NK cell (48). Previously, Varchetta et al. found that trastuzumab therapy caused ADCC of NK and NKT cells in 15 of 18 patients with breast cancer overexpressing Her2, and the killing capacity of CD16^+^ lymphocytes depended on 158 V/F polymorphism of CD16A (49). This study indicates that the effectiveness of short-term trastuzumab monotherapy may be associated with ADCC. However, ADCC is actually influenced by a number of factors. First, the difference in CD16A allotype affinity between cancer patients may contribute to individual heterogeneity of ADCC. Patients with CD16A 158VV alleles showed higher affinity for IgG mAbs than those bearing 158FF and 158VF alleles (49). Second, the quantity of NK cells and NKT cells among peripheral blood lymphocytes count for the intensity of ADCC (49). Different ADCC levels in patients have been shown to correlate to the numbers of effector cells. Third, the IgG subclasses vary in affinity to CD16A and this binding difference decides their capacity to elicit ADCC. It has been demonstrated that IgG3 and IgG1 show high affinity to CD16A, followed by IgG4, while the affinity of IgG2 toCD16A is extremely low (50). Besides, ADCC is associated with the concentration of IgG mAb in a dose-dependent manner. Humoral components may suppress ADCC, considering the potential competition between conventional mAbs and human serum IgG for binding CD16A (51, 52).

Some studies have focused on the modification of mAbs to enhance ADCC by increasing affinity for Fc receptors through mutagenesis or glycosylation. An Fc-engineered CD133 antibody containing the S239D/I332E substitution showed an improved affinity to NK cells and potent NK cell degranulation in a xenograft model of human acute myeloid leukemia (AML) (53). Alternatively, the transgenic chicken derived anti-CD20 mAbs presenting 14 N-glycan patterns such as terminal galactosylation, afucosylation, and high-mannose showed significantly improved Fc-mediated roles, such as ADCC and complement-dependent cytotoxicity (CDC), compared to commercial rituximab, a chimeric mAb against CD20 (54).

Recently, Ferrari de Andrade et al. generated mAb 7C6 against MICA/B (Major Histocompatibility Complex Class-I chain related gene A/B), which could prevent the extracellular domains of MICA and MICB from proteolytic shedding and mediated antitumor immunity by activation of NKG2D and CD16 on NK cells. In a metastasis model pretreated with human NK cells, mAb 7C6 effectively inhibited tumor growth *in vivo* (55).

Immunocytokines are another choice. For instance, hu14.18-IL2 is a humanized mAb that is covalently linked to two molecules of IL-2 at the Fc region. It has been demonstrated that it effectively treats patients with advanced melanoma or neuroblastoma (56).

#### Use of Bi- or Tri-specific Killer Engagers to Potentiate NK Cell Activity

Bi-specific or tri-specific killer cell engagers (BiKEs or TriKEs) are designed moieties containing single-chain variable fragments (scFv) against both TAAs and activating receptors on NK cells to create an immunologic synapse between NK cells and tumor cells (57).

CD16 is an attractive candidate for mediating NK cell-dependent tumor cell killing. It has been demonstrated that CD16-directed bi-specific (CD16×19) and tri-specific (CD16×19×22) scFv agents directly stimulate NK cells via CD16, potentiating NK cell lytic activity and cytokine release to attack lymphoid tumors (58, 59). Another novel BiKE antibody, CD16 × 33 BiKE, signals via CD16 and targets the myeloid differentiation antigen CD33. It could specifically trigger the lytic activity of NK cells and the release of cytotoxic granules to fight against AML cells *in vitro*. Besides, in the same study, it was found that addition of the ADAM17 inhibitor, which prevents CD16 shedding, can enhance the effector roles of NK cells (60). NKG2D, a lectin-type receptor and one of the major activating NK cell receptors, is another powerful candidate for mediating NK cell immune surveillance. A CS1-NKG2D bi-specific antibody, containing an anti-CS1scFv and an anti-NKG2D scFv, displayed a dose-dependent increase in specific cytotoxicity of NK cells as well as cytokine production *in vitro*, and significantly prolonged survival in a human multiple myeloma (MM) model (61).

In recent years, modified killer cell engager antibodies have been developed in terms of design and structure. For instance, AFM13, a tetravalent bi-specific tandem antibody against CD16A and CD30, works in an Fc-independent manner with high affinity without Fc domains binding to CD16A. Due to its molecular weight of 104 kDa (BiKE/TriKE: 50-75 kDa), AFM13 has a longer half-life than other bi/tri specific antibodies. Its tolerability and safety have been tested in relapsed/refractory Hodgkin lymphoma patients, with 77% of subjects showed disease stable (62, 63). It is currently under evaluation in a phase II monotherapy trial in relapsed/refractory Hodgkin lymphoma patients (64). TriKEs are developed on the format of BiKEs. Improvements in newer generation TriKE include promoting specificities to targets, modifying scFvs with high affinity to NK cells, as well as enhancing the self-sustaining of NK cell activity (65). For instance, a modified IL-15 cross-linker has been integrated into TriKE to enhance the survival and expansion of NK cells *in vivo* (66, 67). 161533 TriKE, consisting of anti-CD16 scFv, a modified IL-15 linker and anti-CD33 scFv, could direct antigen specific ADCC while increasing both NK cell survival and proliferation against AML *in vivo* (66). 1615EpCAM TriKE (CD16×IL-15×Epcam) significantly enhanced NK cell proliferation, lytic degranulation and cytokine secretion *in vitro* (67).

Although the BiKE/TriKE therapies have the capability to re-direct NK cytotoxicity, enhance NK function, as well as can be exploited in combination with existing cancer immunotherapies, there are still many obstacles impeding their clinical application. Challenges to successful translation of BiKE/TriKE may include the structure design, such as the identification of TAAs and selection of appropriate joint linker (68, 69). Suitable TAAs could help reduce the off-target effects of these engineered molecules, which is also important for the clinical translation of CAR-T therapy. On the other hand, the joint linkers selected in BiKE/TriKE have the potentials to dominate the biodistribution of entire molecules, which may either increase or decrease their tumor targeting ability. It is reported that cytokine portion can affect the whole immunocytokine molecule to recognize irrelevant antigen, indicating the careful assessment of the biodistribution is crucial for the format design (70). Another challenge is the production technology. Although protein yields have been improved with the development of expression system, cost-efficient and high-yield manufacturing remains a problem (71).

#### Targeting the Immune Checkpoints to Release the Inhibition on NK Cells

The purpose of antibodies targeting inhibitory NK cell receptors, especially immune checkpoint-targets, is to unblock a blocked immune response to increase anti-tumor activity. This is different from NK cell “enhancers” who activate the immune system to improve anti-tumor responses.

One of the main types of inhibitory receptors is inhibitory killer-cell immunoglobulin-like receptors (KIRs), which could be targeted by IPH2101 and IPH2102 (lirilumab) antibodies. IPH2101 and IPH2102 are clinical grade immunoglobulin G4 (IgG4) mAbs which interact with KIR2D moieties. *In vitro*, they augment NK cell-mediated killing against KIR-ligand expressing and antibody coated tumor cells (72, 73). The tolerability and safety of IPH2101 were confirmed by Phase I clinical trials in patients with AML or relapsed/refractory MM (74, 75). However, IPH2101 only induced insufficient clinical response in a Phase II clinical trial in patients with smoldering MM (76). Carlsten et al. demonstrated that IPH2101 infusion led to significant decrease in both KIR2D expression on NK cells and NK cell responsiveness, which might be the underlying mechanism behind the low therapeutic efficacy of IPH2101 (77). The loss of KIR2D on NK cells induced by IPH2101 was mediated by trogocytosis, a mechanism whereby activated neutrophils and FcγRI expressing monocytes extracted KIR2D from the NK cells and expressed this antibody-targeted molecule on their own surface. These findings suggest that the application ofIPH2101-mediated KIR2D blockade *in vivo* might be restricted by the loss of KIR2D on NK cells and subsequent hyporesponsiveness to anti-KIR antibody. As a human IgG4 mAb derived from recombinant chinese hamster ovary cells, IPH2102 (lirilumab) shares the primary amino acid sequence with IPH2101, with the exception of a mutation incorporated at the heavy chain constant region, by the substitution of serine for proline. In a first-in-human, phase I trial using high lirilumab doses in patients with high sensitivity to NK cell modulation, no dose-limiting toxicity recorded (78). Thus, further investigation should examine the efficacy of IPH2101 and IPH2102 in different disease settings as well as their therapeutic value when combined with other therapies, e.g., cytokines or tumor-targeting mAbs.

IPH2201 (humZ270), a mAb bound to NKG2A, showed enhanced NK cytotoxicity to Epstein-Barr virus (EBV) cell lines or engrafted human primary leukemia *in vivo* (79). There are on-going clinical trials aimed at testing its safety, tolerability and efficacy when used singly or when co-administered with other therapies (www.clinicaltrials.gov).

PD-1 is an inhibitory receptor expressed by some activated lymphocytes, including B cells, T cells, and NK cells. PD-1 can be induced on NK cells from cancer patients, and induces functional failure of activated NK cells. This has led to the design of many PD-1 antibodies, including nivolumab, pidilizumab, and pembrolizumab (80). Current research is focused on PD-1 antibodies that can inhibit tumor growth by eliminating T cell immunosuppression, so it is worth exploring the enhanced endogenous NK cell cytotoxicity by PD-1 antibodies.

Another inhibitory receptor found on cell surface of NK cells and T cells is TIGIT. It has been reported that blocking TIGIT is an alternative complementary strategy to the currently available immunotherapies. In a recent study, using tumor-bearing mice, Zhang et al. found that inhibition of TIGIT enhanced NK cell-mediated tumor immunity and blocked NK cell exhaustion, further improving memory responses to tumor re-challenge in an NK cell-dependent way (81). Several other checkpoint proteins including LAG-3(lymphocyte-activation gene 3), TIM-3 and CD96 are also present in NK cells (82).

## Adoptive NK Cell Immunotherapy

Adoptive transfer of NK cells with high yields and high quality is a direct and fundamental approach to replacing, restoring and improving the function of the immune system through infusing allogeneic NK cells activated *ex vivo* or chimeric antigen receptor (CAR) modified NK cells, and has been demonstrated to be a promising cellular immunotherapy against tumor cells (Figure 1B).

### Adoptive Transfer of Unmodified NK Cells

Adoptive transfer of autologous NK cells expanded *ex vivo* for treatment patients with lymphoma, colon cancer, breast cancer and lung cancer have been tested in a range of clinical trials. Only very limited antitumor effect was observed (83–85). The major reason was that the inhibitory receptors on autologous NK cells matched self MHC class I presented on tumor cells, and this “self” recognition signals subsequently inhibited the activation of NK cells (86). Besides, autologous NK cells derived from cancer patients were actually in an immune suppression state with impaired functions, making these cells difficult to exhibit antitumor capability. The first piece of evidence showing that NK cells had a clinical benefit was reported in 2002 (87). It has comfirmed that donor-vs.-recipient NK cell alloreactivity, which was mainly resulted from KIR ligand incompatibility, could avoid relapse and graft rejection without GVHD in AML patients receiving HLA (Human leukocyte antigens) mismatch donor hematopoietic transplantation (87). Later, this strategy was used in adoptive cellular immunotherapy of *ex vivo* activated allogeneic KIR/KIR ligand mismatched NK cells derived from PBMC into AML patients (88). Subsequently, alloreactive PBMC derived NK cells have been widely investigated as an immunotherapy in clinical trials of hematologic malignancies, as well as in trials of solid tumors including melanoma, breast cancer, ovarian cancer, neuroblastoma, renal cell carcinoma, colorectal cancer, and hepatocellular cancer (89, 90). The rules for infusion of allogeneic NK cells as cancer treatment have been reviewed by Leung Wing (91). For manufacturing large numbers of alloreactive NK cells prior to reinfusion, cytokines such as IL-2 and IL-15 were initially used for *ex vivo* culture and expansion of primary NK cells from peripheral blood (PB) (92). However, it was difficult to obtain large numbers of functional NK cells by only exposure to cytokines. Subsequently, artificial antigen-presenting cells (aAPCs) were developed as feeder cells for expanding NK cells *ex vivo*. Our group developed K562-based aAPCs with CD137L-IL-21, and found that they facilitate the log growth phase of NK amplification without indication of senescence *in vitro* culture (Invention patent NO. ZL201110075736.1). Using the aAPCs with CD137L-IL21 as feeder cells, a closed, large-scale and automated bioreactor under GMP conditions can be successfully used for manufacturing large numbers of activated NK cells, which has significantly promoted clinical trials for the application of NK cell-based immunotherapy.

The second way to produce large numbers of cytotoxic NK cells for immunotherapy is through a clonal NK cell line, such as NK-92, KHYG-1, HANK-1, NKG, NK-YS, YTS, YT, NK3.3, and NKL, all of which can expand and propagate easily in culture medium (93). Among them, NK-92 is the only cell line approved by the US FDA for use in the clinical trials, though these EBV-transfected cells must be irradiated before adoptive transfer to prevent propagation in patients (94). NK-92 cells can easily proliferate in flasks, gas-permeable bags, or bioreactors in compliance with GMP, with a doubling time between 24 and 36 h to produce effective clinical-grade NK-92 effectors (93). Although the safety and tolerability of NK-92 cells have been tested in several Phase I clinical trials, limited data exist on their clinical benefits (94–96). In a recent dose escalation trial (NCT00900809), the feasibility of adoptive transfer of NK-92 cells was assessed in 7 patients with refractory/relapsed AML. None of the patients experienced DLT, while no one had complete remission. Besides, no significant changes were observed in either counts or activity of NK cell, CD8+ T cell, CD4+ T cell, T regulatory cell and myeloid-derived suppressor cell (MDSC) (97). It has been indicated that the limited clinical efficiency of NK-92 cells is mainly due to the lack of CD16 expression and their short longevity *in vivo* (32). To overcome these barriers, high affinity natural killer (haNK) cells, a variant of NK-92 cell line engineered to CD16A (158VV) receptor and endogenous IL-2, has been successfully developed. This variant can mediate ADCC as well as bypass the need for exogenous IL-2 in culture (98). The feasibility of haNK cells is currently under investigation in patients with solid tumors (NCT03027128). Moreover, target-activated NK-92 (taNK) cells, another engineered variant of NK-92 cells modified with CARs, have been designed to target TAAs-expressing tumor cells (99, 100). The stable CAR expression and activity of these cells have been demonstrated by HER2.taNK (HER2-specifc target-activated NK), a cell line that is now being tested in patients with recurrent HER2-positive Glioblastoma (NCT03383978).

The third source of NK cells for tumor lysis are NK cells differentiated from human pluripotent stem cells (hPSCs), including hematopoietic stem/progenitor cells (HSPC) from umbilical cord blood (UCB), induced pluripotent stem cells (iPSCs), and human embryonic stem cells (hESCs) (101–103). NK cells derived from hPSCs can be utilized as an allogenic product. The protocols for producing NK cells from hPSCs often involve separating or generating CD34+ hematopoietic precursors and culturing in the presence of NK cell maintaining cytokines (IL-3, IL-7, IL-15, SCF, FLT3L) to further stimulate differentiation and growth of NK cells (104). These NK cells can be expanded by coculturing with aAPCs, which resulted in a 2–3 log expansion that lasted for more than 2 months (105). NK cells differentiated from stem cells have a phenotype close to primary NK cells, a stronger proliferative ability and a homogenous, genetically defined population which meets the requirements of their clinical applications (103, 104, 106). Recently, it has been demonstrated that long-term cryopreservation (1–10 years) had no effect on the expansion potential and effector function of UCB-derived NK cells, which made UCB an emerging source of NK cells. Particularly, there are over 600,000 UCB -units stored globally (107). The safety and tolerability of some UCB-derived NK cells have been tested in phase I clinical trial against AML and myelodysplastic syndromes (108).

In order to obtain stronger tumor killing activity of transferred NK cells, immune-stimulatory molecules such as antibodies and cytokines are often used in combination with NK cells in clinical studies (89, 109). Moreover, considering that NK cells can modulate host immune system against cancers, and hence function as immune adjuvants, they are able to boost the efficacy of anticancer therapy when combined with traditional treatments such as chemotherapy. In several clinical trials on NK cell-based immunotherapy, patients received chemotherapy prior to NK cell infusion (www.clinicaltrials.gov). Particularly, several drugs used in cancer chemotherapy could stimulate expression of activating ligands on tumor cells, which makes tumor cells more sensitive to NK cell-mediated cytotoxicity. Some of these drugs may also promote the recruitment of NK cells to the tumor sites by increasing secretion of selected chemokines (110). Among them, lenalidomide seems to be the most active drug which overcomes suppression of human NK cell anti-tumor functions by TME-associated IL-6 and TGF-β and lowers the threshold of MICA for NKG2D-mediated NK cell activation and augments nanoscale rearrangements in cortical actin at the NK cell ADCC immune synapse (111, 112). In clinical settings, the clinical trials of expanded and activated NK cell adoptive transfer are always in conjunction with lenalidomide treatment (NCT02573896, NCT02280525, NCT02481934, NCT02525250).

### Adoptive Transfer of CAR-NK Cells

Unlike immunostimulatory strategies which involve the delivery of molecules to assist NK cells, genetic modification strategies induce changes in the genetics of NK cells directly, leading to far-reaching and sustained changes to the cells (113). Among them, genetic modification of NK cells with CAR constructs has drawn increasing attention. CAR-NK cells can be produced from different sources of NK cells including primary NK cells, NK cell lines, and HPSCs. CAR-NK has adopted the basic structural framework of CAR-T, that is, chimeric antigen receptors mainly composed of extracellular, hinge, transmembrane and intracellular domains, as well as the transfection methods (100, 114, 115). The extracellular domain can bind tightly to tumor-associated antigens expressed on the surface of tumor cells, which determines the specificity of CAR structures. Single-chain variable fragments (ScFvs) are the most commonly used ectodomains for CARs. The hinge domain is the connecting sequence between the extracellular domain to the transmembrane domain, which endowed CAR with sufficient orientation and flexibility to bind to tumor antigens and are expected to impact the CAR-NK activities. The transmembrane domain lies between the hinge and the intracellular signaling domain, including CD3ζ, HLA-A2, or CD28 molecules. The structure of the intracellular signaling domain determines the intensity of the CAR-NK activation signal, which contains the immunoreceptor tyrosine-activated motifs (ITAMs). The majority of current CAR endodomains contain an activation region derived from CD3ζ, which is the most classical intracellular domain including three ITAMs. In order to increase the proliferation and cytotoxicity of CAR modified effector cells, co-stimulatory protein receptors such as CD28, 4-1BB, CD134, ICOS are added to the cytoplasmic tail.

The first CAR used in NK cells is a CD4-CD3ζ (CD4ζ) fusion receptor, which was reported by Tran et al. (116). In this study, the CD4ζ chimeric receptor is biochemically and functionally active and can guide human NK cells efficiently to kill either HIV-infected CD4^+^ T cells or NK-resistant tumor cells expressing gp120 *in vitro*, indicating CAR structure can be successfully expressed on NK cells, leading to efficient retargeting (116). Subsequently, more attempts were taken to enhance the anti-tumor ability of NK cells. Uherek et al. modified NK-92 cells with ErbB2-specific CAR, and found these cells show specific cytotoxicity in animal models (117). While, Imai et al. evaluated the efficacy of primary NK cells expressing CD19 CAR against autologous leukemic cells *in vitro*, and demonstrated these cells can bypass inhibitory signals (118). To date, CAR-NK cells have been evaluated for the treatment of hematological cancers and solid tumors in preclinical studies with numerous good results [reviewed in (32, 36, 100)]. On the basis of these findings, the clinical translation of CAR-NK cells has witnessed significant interests (see Table 1 for details of ongoing clinical trials). Although CAR-NK therapy is still under clinical evaluation, NK cells possess several advantages over T cells in being engineered to express CARs and used for cancer treatment. First, NK cells are easy to be isolated and have a relatively short lifespan. Therefore, the risk of overexpansion of transferred CAR-NK cells in patients is relatively low. Second, the cytokines secreted by NK cells mainly include IFN-γ and GM-CSF, which are relatively safer than those released by activated CAR-T cells, e.g., TNF-α and IL-6. In particular, the proinflammatory cytokines produced by CAR-T cells may cause life-threatening cytokine release syndrome (CRS), a most common and severe side effect of CAR-T therapy (119). Third, CAR-NK cells could trigger the lysis of target cells in both CAR-dependent and CAR-independent manners, which further potentiating their killing activity. In addition, CAR-NK therapy is expected to be less expensive, considering that NK cells can be derived from PBMCs, NK cell lines and hPSCs. However, T cells used for CAR-T therapy are required to be autologous (119, 120).

**Table 1:** Current clinical trials of CAR-NK cells.

**ClinicalTrials.gov Identifier**	**Trial**	**Status**	**Phase**	**NK source**	**Target**	**Signaling domain of CAR**	**Institute**
NCT02742727	CAR-pNK cell immunotheray in CD7 positive leukemia and lymphoma	Recruiting	I/II	NK-92	CD7	CD28-4-1BB-CD3ζ	PersonGen BioTherapeutics (Suzhou) Co., Ltd. Suzhou, Jiangsu, China
NCT02892695	PCAR-119 bridge immunotheray prior to stem cell transplant in treating patients with CD19 positive leukemia and lymphoma	Recruiting	I/II	NK-92	CD19	CD28-4-1BB-CD3ζ	
NCT02839954	Study evaluating the efficacy and safety of chimeric antigen receptor-modified pNK cells in MUC1 positive advanced refractory or relapsed Solid Tumor	Recruiting	I/II	NK-92	MUC1	–	
NCT03383978	Study of intracranial injection of NK-92/5.28.z (HER2.taNK) cells in patients with recurrent HER2-positive Glioblastoma	Recruiting	I	NK-92	HER2	CD28-CD3ζ	Johann W. Goethe University Hospital, Frankfurt, Germany
NCT03056339	Dose escalation study phase I/II of umbilical cord blood-derived CAR-engineered NK cells in conjunction with lymphodepleting chemotherapy in patients With relapsed/refractory B-Lymphoid malignancies	Recruiting	I/II	HSPC	CD19	CD28- CD3ζ	University of Texas MD Anderson Cancer Center Houston, Texas, United States
NCT03579927	Combined therapy of CAR.CD19- CD28-CD3ζ-2A-iCasp9-IL15-transduced cord blood NK cells, high-dose chemotherapy, and stem cell transplant in Treating participants with B-cell lymphoma	Not yet recruiting	I/II	HSPC	CD19	CD28- CD3ζ	
NCT01974479	Pilot study of redirected haploidentical natural killer cell infusions for b-lineage acute lymphoblastic leukemia	Suspended	I	Primary NK cells	CD19	4-1BB-CD3ζ	National University Health System, Singapore
NCT03415100	Pilot Study of NKG2D-ligand targeted CAR-NK cells in patients with metastatic solid tumors	Recruiting	I	Primary NK cells	NKG2D	–	Third Affiliated Hospital of Guangzhou Medical University Guangzhou, Guangdong, China

Despite of the significant advantages and great potentials, there are still challenges that should be addressed carefully to prevent hindering the clinical application of CAR-NK therapy. First, design of the optimal structure of CARs on NK cells has not been systematically studied yet. The position of the CAR-binding epitope and its distance to the CAR-NK cell surface are expected to affect the binding to the antigens, the optimal formation of immune synapses and CAR-NK cells activation (100). Empirical observations from CAR-T cells indicate that the structure of the hinge and transmembrane domains can affect CAR specificity and activity. In addition, there is a differential requirement for costimulatory signals between the established NK-92 cell line, primary NK cells and hPSC-derived NK cells. The study with three kind CD19-specific NK-92 cells, whose CARs harbor the same anti-CD19 scFv and hinge region, but linked to human CD3ζ, composite CD28-CD3ζ or 4-1BB-CD3ζ extracellular domains, respectively, showed that 4-1BB-CD3ζ-based CD19-specific NK-92 cells were less effective than CD3ζ or CD28-CD3ζ in cytotoxicity and cytokine production (121, 122). While for CD19-specific primary NK cells expanded *ex vivo*, inclusion of 4-1BB or 2B4 costimulatory domains together with CD3ζ was shown to promote both cytokine production and specific cytolysis (116, 123). It is also notable that, recently, Li et al. expressed CAR constructs which were specifically tailored to promote NK cell activation in iPSC-derived NK cells. They showed that such constructs containing the transmembrane NKG2D and intracellular 2B4 domains exhibit superior performance compared to CAR constructs designed for T cells (124). Given the unique activating receptor repertoire and signal-activating proteins of NK cells, customizing NK-based CAR structures will have great potential for improving CAR-NK function.

Second, although optimization of transduction methods in the last few decades has led to higher efficiencies, retroviral transduction is dependent on the existence of an active cell division, which weakens the efficiency of this strategy with non-activated NK cells. Genotoxicity of insertional mutagenesis is still a potential safety concern for lentiviral vectors. Furthermore, NK cell death associated with these procedures has limited the use of genetically engineered NK cells. Only few investigations have documented results regarding the capability of NK cells to function successfully after viral transduction. The innate properties that characterize NK cells may result in their reduced efficacy after viral transduction and apoptosis. As part of the immune systems innate defense mechanisms, pattern recognition receptors (PRRs) are used to identify pathogen associated molecular patterns (PAMPs). These procedures are likely involved in triggering a cascade of cellular events that lead to apoptosis of NK cells after viral transduction (125). Nevertheless, RNA engineering gave NK cells higher survival rates and transfection efficiency compared to viral gene transfer. One study found that NK cells differentiated from gene modified human hematopoietic stem cells have high gene transfer efficiency and viability of CAR-NK cells (126).

Third, albeit the primary targets of CARs are antigens expressed on abnormal cells, most currently available CARs are directed against non-mutated autoantigens that are differentially expressed on cancer cells. This may result in non-specific binding of CARs to antigens expressed on normal tissues. Therefore, antigen-positive healthy tissues are at risk of accidental injury induced by off-target activity. Anti-CD19 T cell therapy for B cell malignancies has shown strong off-target toxicity against normal CD19-positive B cells (127). There is no clinical data to assess the extent of off-target toxicity of CAR-NK, but identification of specific tumor targets is the basis for a safe and effective CAR construct. To increase the specificity and safety of CAR molecules, the experience from CAR-T studies can be useful. Effective reduction of cytotoxicity occurs if two individual structures, or two extracellular domains, are combined in a tandem form to produce a bi-specific CAR molecule (128–130). Importing a suicide gene, such as inducible caspase-9 (iCasp9) (131), facilitates control of dosage for CAR activity and provides another strategy to mitigate toxicity. A similar concept was described with a split CAR construct that requires dimerization of a small molecule to form a functional unit, further increasing safety (132).

Another safety concerning is that the infused expanded allogenic NK cells derived products may be contaminated by T or B cells, theoretically resulting in GVHD or post-transplant lymphoproliferative disorders, respectively. One study reported that donor-derived, IL-15/4–1BBL-activated NK cells infused early after major histocompatibility–matched, T-cell–depleted peripheral blood stem cell transplantation in patients could cause acute GVHD (133). They also demonstrated that GVHD was highly frequent in matched unrelated donor transplants and linked to higher CD3 chimerism. This later aspect refers to the establishment of an optimal clinical-grade protocol for purification of activated CD56^+^CD3^−^ NKs. This protocol could be safer for patients even in the presence of transfused third party NK cells modified with CARs. In any case, it is encouraging to note that clinical studies of haploidentical and UCB-derived NK cell infusions in hundreds of patients with both hematologic and solid malignancies have not reported a higher risk of GVHD (32).

## Cell-free Derivatives From NK Cells for Cancer Immunotherpy

Currently, cancer immunotherapy strategies are dominated by immune cells. However, there are limitations when using immune cells directly for the treatment of cancers. For instance, immune cells can hardly penetrate into solid tumor, leading to unsatisfactory therapeutic effects. Moreover, the cost for producing, preserving and transporting clinical grade living immune cells is high, posing a challenge to wide applications in clinics. In recent years, EVs, a nano-sized vesicles naturally secreted by many different types of cells including NK cells, have gradually been proposed and studied, providing a new cell-free immunotherapy avenue (Figure 1C).

### Biology of NK EVs

**Figure 2:**
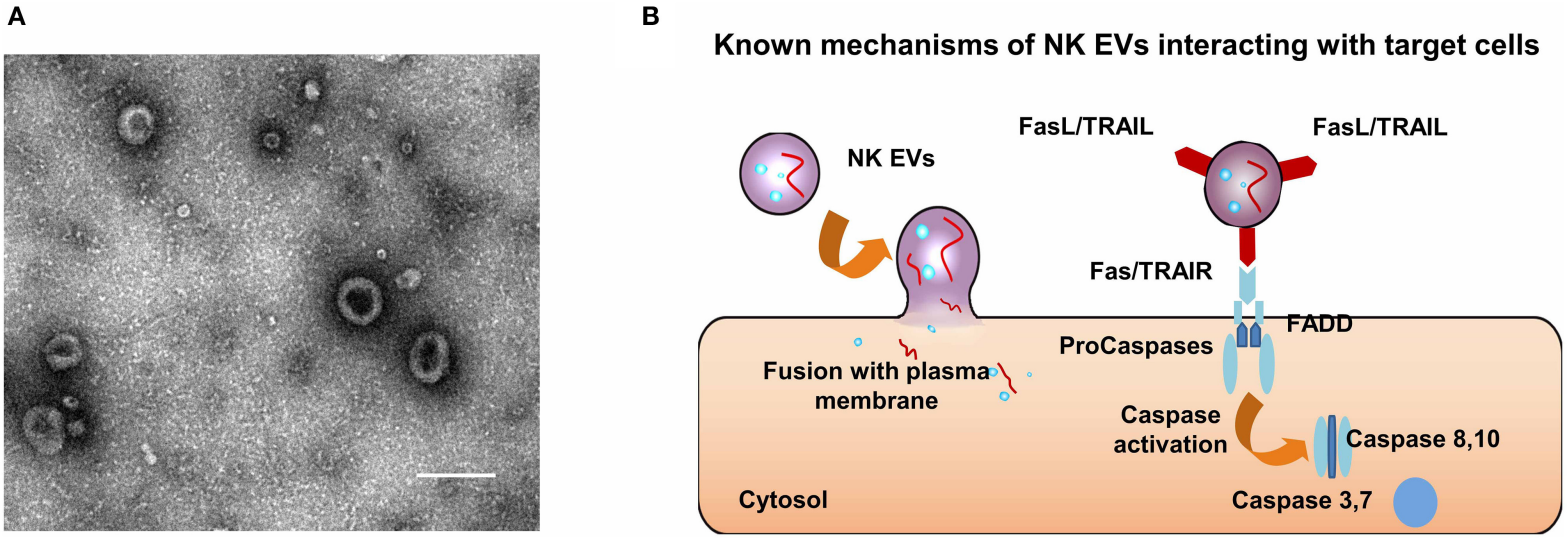
Characteristics and anti-tumor mechanisms of NK EVs. **(A)** A typical micrograph of transmission electron showing extracellular vesicles obtained from NK cells; scale bar, 200 nm. **(B)** Mechanisms by which NK EVs may interact with target cells.

EVs are used to depict three types of vesicles with lipid bilayer membrane. Exosomes are the smallest membrane vesicles (40–100 nm) that are secreted by most cell types by multi-vesicular bodies (MVBs) fusing with the cell membrane. Microvesicles (MV, 50 nm-1 μm) are vesicles that a little larger than exosomes, budding outwards from the plasma membrane. Apoptotic bodies (50 nm-5 μm) are the third kind of EVs, which are generated from cells undergoing apoptosis, by an outward blebbing way. In this review, we defined “EVs” as small extracellular vesicles with nanometer size. In transmission electron microscopy images, EVs usually appear as cup-shaped entities (Figure 2A). EVs express both common biomarkers, such as tumor susceptibility gene 101 protein (TSG101) and tetraspanins (CD9, CD63, CD81, and CD81), and specific proteins based on their cell origin (134). For instance, EVs derived from human NK cells (NK EVs) contain not only typical NK markers (e.g., CD56), but also lytic proteins (e.g., FasL, perforin).

EVs are involved in a number of key cellular processes due to their ability to transfer bioactive cargos (i.e., nucleic acids, lipids, and proteins) to recipient cells or by activating signaling pathways in target cells (135–138). Therefore, they are considered as potential alternatives to cell-based therapeutics, with characteristics possibly superior to cells in some aspects. First, since EVs are nano-sized, they can diffuse through tissues passively, partly due to the leaky vasculature of the tumor and subsequent enhanced retention effect (139). Second, although the acidic environment of solid tumors caused by hypoxia-ischemia increases immunosuppression (140, 141), it is beneficial to promote the fusion between EVs and tumor cells (142). Third, these “nano bullets” have an advantage in their ability to traverse biological barriers such as the blood-brain barrier (BBB) and the blood-tumor barrier (BTB), which has provided a completely different methodology compared to the cell-based approach (143–145). Moreover, EVs are stable at −80°C, lasting up to 12 months (146). This makes EVs easy to store and readily accessible as “off-the-shelf” supply.

### Anti-tumor Activity of NK EVs

The first study on NK EVs study was conducted by Lugini et al. (147). In this study, it was demonstrated that isolated NK EVs from purified NK cell culture exerted a cytotoxic effects on malignant hematologic cell lines, but not on solid tumor cells like breast carcinoma SKBR3 (147). Later, activated NK EVs, obtained from large scale isolation of *in vitro* expanded NK cells, showed significant cytotoxic effects on a series of cancer cell lines, including ALL, neuroblastomas, and breast carcinomas (146). These findings suggest that activated NK cells may produce EVs with higher immune activity compared with naïve NK cells. The antitumor effect of NK EVs is further supported by two recent *in vivo* studies. In one study, NK EVs were isolated by density gradient ultracentrifugation from NK-92 cells, and then injected into melanoma locally in a mice model. As a result, the progression of tumors treated with NK-92 EVs were significantly inhibited (148). In another study conducted by the same group, NK EVs prepared in the same way were used for the treatment of glioblastoma xenograft tumors. The intravenous administration of NK EVs not only significantly suppressed tumor growth, but also exhibited tumor-specific accumulation and an ability to cross the BBB. Encouragingly, this tumor-specific accumulation persisted for more than 5 days (149).

Fais et al. reported that NK EVs could trigger the death of target cells by two distinct mechanisms (137, 150), including ligand-receptor interactions (151, 152) and plasma membrane fusion (142) (Figure 2B). Furthermore, caspases, the initiator and executioner, were involved in the cell killing mediated by NK EVs (Figure 2B). Wen et al. extrapolated that the killing mechanisms of NK EVs can be classified into three groups, including perforin-granzyme mediated entry, receptor-ligand mediated interaction and granulysin mediated action (153). However, the underlying mechanisms of specific killing of tumor cells mediated by NK EVs remains unclear. NK cells recognize target cells for killing by a “self-missing” mechanism, but there is no evidence to prove that EVs can activate signaling pathways like living immune cells do. One possible reason why NK EVs can have a targeting effect is that the acidic microenvironment of solid tumors can promote the fusion of exosomes. In the study by Lugini et al. mentioned above, the secretion of NK EVs was stable and had a killing effect on activated PBMC cells *in vitro*, suggesting that NK EVs behave like a large “reservoir” to regulate the balance of immune cells (147). This mechanism is still not being clearly delineated. However, its potential for application in autoimmune diseases that result from excessive immune activation is obvious, as well as its potential use for elimination of side effects of immunotherapy such as cytokine storms is also worth exploring.

Notably, even though their anti-tumor activity has been demonstrated *in vitro* and *in vivo*, NK EVs have no impact on normal cells (147). In addition, due to the protective barrier offered by a natural vesicle structure, NK EVs might be used as a promising delivery system, which can be loaded with various therapeutic substances, e.g., genes and proteins. Particularly, the surface of EV membrane can be further modified by other functional groups.

## Perspective

Cancer immunotherapy strategies are evolving around different immune cells. Although the developments in the field used to be slow, a rising number of approaches are being actively exploited to boost NK cell-based anti-tumor immune functions. Immune-stimulatory molecules can either selectively enhance NK cell activity while maintaining their *in vivo* survival and proliferation (e.g., cytokines), or mediate NK cell cytotoxicity by ADCC (e.g., antibodies). With the advances in *ex vivo* expansion/activation technology, as well as new approaches to genetically modify NK cells, adoptive transfer of NK cells holds promises to become powerful weapons in the fight against cancers. Moreover, EVs derived from NK cells have become a rising star and caught more and more attentions. Because of their nanometer size and the propensity to travel and survive in the acidic tumor microenvironment, NK EVs might be potent for the treatment of solid tumors. In particular, the homologous nature of NK cells provides great advantages for the clinical translation of NK-cell based therapeutic approaches.

## Author Contributions

All authors were involved in the concept and design of the manuscript, search, and analysis of the literature. WH and GW drafted the initial version of the manuscript with support from all other co-authors. All authors revised and approved the final version of the manuscript.

### Conflict of Interest Statement

The authors declare that the research was conducted in the absence of any commercial or financial relationships that could be construed as a potential conflict of interest.

## Abbreviations

NK: Natural killer
CARs: Chimeric antigen receptors
EVs: extracellular vesicles
NKG2D, Natural-killer Group 2: Member D
TME: Tumor microenvironment
DCs: Dendritic cells
Treg: Regulatory T
TGF-β: Transforming growth factor-β
BM: Bone marrow
PB: Peripheral blood
IFNγ: Interferon gamma
KIR: Killer-immunoglobulin-like receptors
CTLA-4: Cytotoxic T-lymphocyte-associated protein 4
PD-1: Programmed death-1
Ig: Immunoglobulin
TIGIT: T cell immunoreceptor with Ig and ITIM domains
TIM-3: T cell Ig and mucin domain containing-3
DNAM-1: DNAX Accessory Molecule-1
DR: Death receptor
ADCC: Antibody-dependent cell-mediated cytotoxicity
IgG: Immunoglobulin G
TAA: Tumor-associated antigen
MHC: Major histocompatibility complex
IL: Interleukin
APC: Antigen receptor cell
rh: recombinant human
MTD: maximum tolerated dose
allo-HCT: allogeneic hematopoietic cell transplantation
DLT: dose-limiting toxicities
GVHD: Graft-vs.-host disease
BCG: Bacillus Calmette Guerin
AML: Acute myeloid leukemia
EBV: Epstein-Barr virus
mAb: Monoclonal antibody
CDC: Complement-dependent cytotoxicity
MICA: Major Histocompatibility Complex Class-I chain related gene A
MICB: Major Histocompatibility Complex Class-I chain related gene B
BiKE: Bispecific killer engager
TriKE: trispecific killer engager
ScFv: Single chain antibody fragment
MM: Multiple myeloma
LAG-3: Lymphocyte-activation gene 3
aAPCs: Artificial antigen-presenting cells
FDA: Food and Drug Administration
PBMC: Peripheral blood mononuclear cells
MDSC: myeloid-derived suppressor cell
HPSC: Human pluripotent stem cell
HSPC: Hematopoietic stem/progenitor cells
UCB: Umbilical cord blood
iPSC: Induced pluripotent cells
hESCs: human embryonic stem cells
ScFvs: Single-chain variable fragments
HLA-A2: Human leukocyte antigens-A2
ITAMs: immunoreceptor tyrosine-activated motifs
PRR: Pattern recognition receptor
PAMP: Pathogen associated molecular patterns
iCasp9: Inducible caspase-9
GvHD: Graft-vs.-host disease
MVB: Multi-vesicular body
TSG101: Tumor susceptibility gene 101 protein
BBB: Blood-brain barrier
BTB: blood-tumor barrier
GMP: Good manufacturing practices.
